# Gas Gangrene as a Result of Femoral Traction Pin Placement

**DOI:** 10.1155/2011/459812

**Published:** 2012-01-15

**Authors:** Benjamin C. Taylor, Thomas J. Bramwell, Nathan Formaini

**Affiliations:** Department of Orthopaedic Surgery, Grant Medical Center, 285 East State Street, Suite 500, Columbus, OH 43215, USA

## Abstract

Treatment of adult femoral shaft fractures typically involves operative stabilization with intramedullary implants, external fixation, or a plate and screw construct. However, when stabilization is delayed for any reason, use of a traction pin is recommended to stabilize the fracture, prevent significant shortening, as well as to help with pain control. In this paper, we present the rare complication of a severe gas gangrene infection caused by *Clostridium perfringens* that led to several amputations and ultimately death. We also discuss risks of temporary skeletal traction and techniques to overcome the morbidity of such a procedure.

## 1. Introduction

Placement of a traction pin for temporary stabilization of femoral shaft fractures is an established method of temporarily stabilizing the injury while minimizing fracture shortening and patient discomfort [1, 2]. Traction pins may be placed either in the distal femur or proximal tibia, with different anatomic structures at risk with each [2]. Placement of these pins, although straightforward, has been associated with injury to neurovascular structures, iatrogenic fracture creation, physeal injury, ligamentous injury, and pin site infection [2]. To our knowledge, no report of gas gangrene infection has been reported as a complication of percutaneous temporary skeletal traction.

## 2. Case Report

**Figure 1:**
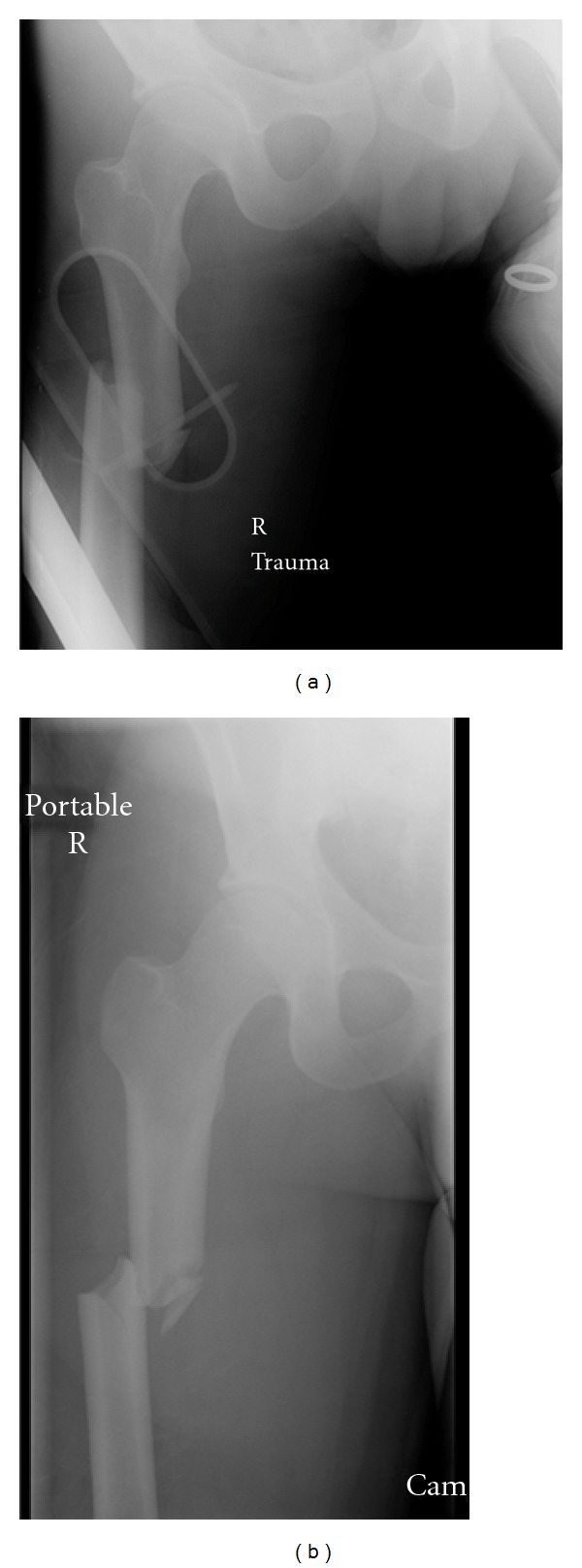
(a) Anteroposterior radiograph of closed right proximal femoral fracture, (b) anteroposterior radiograph of fracture with distal traction applied.

A 21-year-old male was involved in a high-speed motor vehicle collision and sustained multiple injuries, including a right closed subtrochanteric femur fracture, bilateral pulmonary contusions, as well as a splenic injury requiring an exploratory laparotomy with splenectomy upon arrival to the hospital. Due to hemodynamic instability, a distal femoral traction pin with 25 pounds of weight was placed in his right femur on the date of admission as a temporary stabilization of his fracture. This smooth traction pin was placed without difficulty using sterile technique and was placed from medial to lateral at the level of the adductor tubercle. The pin sites were then covered with a sterile dressing. Radiographs of his fracture are shown in Figure 1.

**Figure 2:**
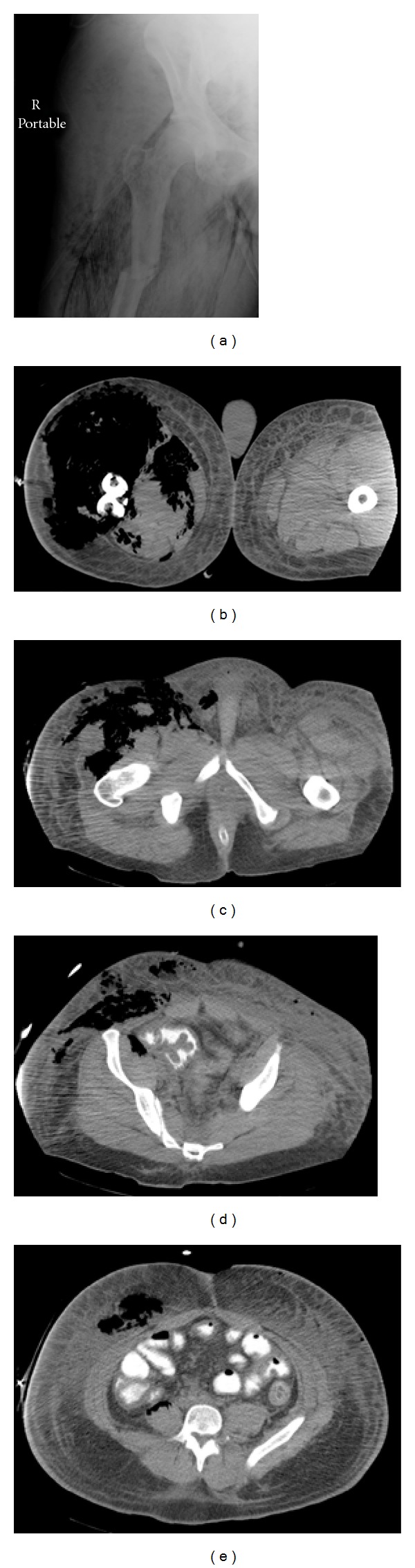
(a) Anteroposterior radiograph of proximal femoral fracture showing a significant amount of soft tissue gas, (b) computed tomography axial slice at the fracture site, showing significant, near-circumferential soft tissue gaseous necrosis, (c) a more proximal axial computed tomography cut showing the significant amount of anterior thigh and hip gaseous necrosis, (d) another more proximal axial computed tomography cut showing evidence of gaseous necrosis along the iliopsoas and anterior abdominal wall, and (e) this axial computed tomography cut shows the most cephalad extension of the soft tissue infection along the anterior abdominal wall.

The patient's overall condition worsened over the ensuing three days, with development of high fevers, increasing leukocytosis, and continued cardiopulmonary lability. He remained intubated throughout his hospitalization. Due to significantly increased swelling and erythema about the right thigh and hip region, a plain radiograph as well as computed tomography scan was ordered to evaluate for abscess or hematoma formation. Representative images from the scan are shown in Figure 2. Mild serous drainage was seen about the traction pin sites, but no purulence was noted. The edema and erythema in the region of the knee was significant as well and was slightly more than the proximal thigh.

 Due to the findings of a significant amount of soft tissue gas in his thigh extending to the groin and caudal abdominal wall, the patient was taken emergently to the operating room for evaluation and debridement. Upon incision into the thigh, malodorous gas was released, and it was discovered that all of the distal muscle and fascia were necrotic; due to these findings, the decision was made to undergo a proximal open guillotine transfemoral amputation through his fracture site. He also underwent debridement of his lower abdominal wall at this time, with removal of all necrotic tissues. Intraoperative deep tissue specimens were obtained at this time and identified *Clostridium perfringens *as the culprit. Unfortunately, the infection continued to spread, and he underwent a hip disarticulation with further abdominal and groin debridement the following day. A final debridement of his hip musculature, including removal of his ipsilateral testicle, was performed two days after his initial amputation. Unfortunately, due to further development of intra-abdominal infection in the face of continued cardiopulmonary instability, the decision was made by family to withdraw support; he ultimately passed away eight days after his injury. 

## 3. Discussion

Temporary treatment of femoral shaft fractures with distal femoral or proximal tibial traction pins is an established method of maintaining overall limb length and minimizing patient discomfort [1, 2]. Localized infection has been reported as a consequence of pin placement, with an increasing incidence as length of pin presence increases [2–4]. However, infection from a traction pin is typically cellulitis or, less commonly, a subcutaneous abscess. Treatment for such an issue requires removal of the traction pin with systemic antibiotics; operative debridement is rarely required. Although definitive treatment of a femoral fracture with traction or even external fixation does carry a risk of deep infection requiring operative debridement [5, 6], to our knowledge, no reports exist of a pin being in place for only five days and becoming so severely infected.

 This patient initially underwent a very proximal open amputation, with further debridement of his anterior abdominal wall and groin on the fifth day after presentation. No further necrotic or questionable appearing material was present at the end of the first debridement, but at his planned return to the operating room the following day, further evidence of soft tissue necrosis was present, requiring hip disarticulation. Although it is likely that a hip disarticulation at the time of original amputation would have decreased bacterial load further, it would have ultimately not made a difference with his eventual mortality, as the infection was evident in his abdominal region at repeat evaluation. At the final debridement of his hip region, only minimal further necrosis was noted, and the development of abdominal infection was the key factor in his demise.

 This patient did not have an open injury, and his soft tissue injury was mild overall, with only superficial contusion and moderate local edema about the fracture site. No penetrating injuries were seen about the extremity or abdomen, and we believe that the etiology of the gas gangrene was ultimately related to the traction pin insertion. Although this was placed using sterile technique, it is possible that the *Clostridium* may have been on the pins if improperly sterilized or may have been present as an environmental contaminant while he resided in the intensive care unit.
